# Periosteal CD68^+^F4/80^+^ Macrophages Are Mechanosensitive for Cortical Bone Formation by Secretion and Activation of TGF‐*β*1

**DOI:** 10.1002/advs.202103343

**Published:** 2021-12-02

**Authors:** Ruoxian Deng, Changwei Li, Xiao Wang, Leilei Chang, Shuangfei Ni, Weixin Zhang, Peng Xue, Dayu Pan, Mei Wan, Lianfu Deng, Xu Cao

**Affiliations:** ^1^ Department of Orthopaedic Surgery The Johns Hopkins University School of Medicine Baltimore MD 21205 USA; ^2^ Department of Biomedical Engineering The Johns Hopkins University Baltimore MD 21205 USA; ^3^ Shanghai Key Laboratory for Prevention and Treatment of Bone and Joint Diseases Shanghai Institute of Traumatology and Orthopaedics Ruijin Hospital Shanghai Jiaotong University School of Medicine Shanghai 200025 China

**Keywords:** cortical bone formation, macrophage, mechanical loading, periosteum

## Abstract

Mechanical force regulates bone density, modeling, and homeostasis. Substantial periosteal bone formation is generated by external mechanical stimuli, yet its mechanism is poorly understood. Here, it is shown that myeloid‐lineage cells differentiate into subgroups and regulate periosteal bone formation in response to mechanical loading. Mechanical loading on tibiae significantly increases the number of periosteal myeloid‐lineage cells and the levels of active transforming growth factor *β* (TGF‐*β*), resulting in cortical bone formation. Knockout of *Tgfb1* in myeloid‐lineage cells attenuates mechanical loading‐induced periosteal bone formation in mice. Moreover, CD68^+^F4/80^+^ macrophages, a subtype of myeloid‐lineage cells, express and activate TGF‐*β*1 for recruitment of osteoprogenitors. Particularly, mechanical loading induces the differentiation of periosteal CD68^+^F4/80^−^ myeloid‐lineage cells to the CD68^+^F4/80^+^ macrophages via signaling of piezo‐type mechanosensitive ion channel component 1 (Piezo1) for TGF‐*β*1 secretion. Importantly, CD68^+^F4/80^+^ macrophages activate TGF‐*β*1 by expression and secretion of thrombospondin‐1 (Thbs1). Administration of Thbs1 inhibitor significantly impairs loading‐induced TGF‐*β* activation and recruitment of osteoprogenitors in the periosteum. The results suggest that periosteal myeloid‐lineage cells respond to mechanical forces and consequently produce and activate TGF‐*β*1 for periosteal bone formation.

## Graphical abstract

Mechanical loading induces the increase in periosteal CD68^+^F4/80^−^ myeloid‐lineage cells, which further differentiate into CD68^+^F4/80^+^ macrophages with the synthesis of Csf1 via Piezo1 signaling. Periosteal CD68^+^F4/80^+^ macrophages secrete TGF‐*β*1 and activate TGF‐*β*1 by producing Thbs1. Active TGF‐*β*1 recruits osteoprogenitors to the periosteal bone surface for cortical bone formation.

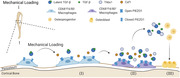

## 1 Introduction

The skeleton provides mechanical support for locomotion, protects vital organs, and regulates calcium and mineral metabolism. Mechanical force is essential to maintain skeletal growth and homeostasis.^[^ ^1^ ^]^ Bone can adapt its architecture and mass in response to mechanical stimuli, such that new bone is formed where force is applied as evidenced by bone gain in athletes.^[^ ^2^ ^]^ Without mechanical loading, bone mass declines, as seen in patients on long‐term bedrest^[^ ^3^ ^]^ and in astronauts who have experienced microgravity.^[^ ^4^ ^]^

Bone has cortical and trabecular compartments. The cortical bone is the compact outer layer covered by periosteum, a thin connective sheath, and protects the trabecular bone in the internal cavity.^[^ ^5^ ^]^ It makes up 80% of human bone mass^[^ ^6^ ^]^ and carries a larger share of load‐bearing than trabecular bone does.^[^ ^7^ ^]^ Repetitive mechanical loading, in turn, has been shown to stimulate cortical bone outgrowth.^[^ ^8^ ^]^ Although cortical bone formation is believed to be a result of cellular events induced by mechanical stimuli,^[^ ^9^ ^]^ the underlying mechanism is unclear.

Growing evidence indicates that the periosteum is responsible for cortical bone development and modeling under mechanical stimulation.^[^ ^10^ ^]^ Periosteum contains different types of cells, from myeloid‐lineage cells (MCs) to mesenchymal‐lineage cells, blood vessels, and abundant matrix proteins for osteogenesis.^[^ ^11^ ^]^ Macrophages, one type of MCs, have been reported to regulate tissue homeostasis and regeneration in specific microenvironments.^[^ ^12^ ^]^ In bones, resident macrophages are traditionally considered to be tartrate‐resistant acid phosphatase positive (TRAP^+^) multinuclear osteoclasts with a primary function of bone resorption.^[^ ^13^ ^]^ Our recent study uncovered that periosteal TRAP^+^ mononuclear cells secrete platelet‐derived growth factor‐BB (PDGF‐BB) to recruit Nestin^+^ and Leptin receptor^+^ (LepR^+^) periosteum‐derived cells (PDCs) to the bone surface. The recruited PDCs undergo osteoblast differentiation for periosteal bone formation coupled with angiogenesis.^[^ ^10c^ ^]^ Moreover, osteal macrophages, a subtype of macrophages, have been shown to support osteoblastic bone formation, bone remodeling,^[^ ^13^ ^]^ and regeneration.^[^ ^12^, ^14^ ^]^ Recent studies have shown that MC functions are related to mechanical stress.^[^ ^15^ ^]^ It has also been reported that MCs sense mechanical forces by the expression of piezo‐type mechanosensitive ion channel component 1 (Piezo1),^[^ ^16^ ^]^ a nonselective Ca^2+^‐permeable cation channel that is expressed in various nonsensory tissues to transmit mechanical signals.^[^ ^8^, ^16^, ^17^ ^]^ However, the role of macrophages in mechanical force‐induced periosteal bone formation has not been well studied.

We^[^ ^18^ ^]^ and other groups^[^ ^19^ ^]^ have found that macrophages highly express TGF‐*β*1 in various tissues, which is essential for bone remodeling through recruiting mesenchymal stem cells and osteoprogenitors.^[^ ^20^ ^]^ TGF‐*β*1 is secreted primarily as an inactivated latent complex and deposited in the extracellular matrix. Additional processes are required to release active TGF‐*β*1, enabling signal transmission to the nucleus by phosphorylating Smad2/3 for further biological functions.^[^ ^21^ ^]^ Typically, the activation of TGF‐*β*1 requires the release of the large latent complex from the matrix followed by additional proteolysis^[^ ^22^ ^]^ or deformation^[^ ^23^ ^]^ of the latency‐associated peptide (LAP). Interestingly, macrophages have been reported not only to secrete TGF‐*β*1 but also to activate TGF‐*β*1 by releasing thrombospondin‐1 (Thbs1) and plasmin.^[^ ^24^ ^]^ Thbs1 is a multifunctional secreted glycoprotein that is expressed widely in the injury and wound healing processes.^[^ ^25^ ^]^ The sequence lysine‐arginine‐phenylalanine‐lysine (KRFK) of Thbs1 recognizes the conserved sequence leucine‐serine‐lysine‐leucine (LSKL) in LAP and disrupts the binding of mature TGF‐*β*1 and LAP. This process generates a conformational change and releases mature TGF‐*β*1.^[^ ^23^, ^26^ ^]^

In this study, we investigated the effect of macrophages on mechanical loading‐induced periosteal bone formation. Our results showed that periosteal macrophages and active TGF‐*β*1 were increased at the site of bone formation following mechanical loading. Macrophages sensed the mechanical signals through Piezo1 ion channel and secreted TGF‐*β*1. Furthermore, macrophage activated TGF‐*β*1 via Thbs1 signaling, which initiated anabolic responses to mechanical loading. The inhibition of Thbs1 diminished the loading‐induced high expressions of active TGF‐*β*1 and osteoblast‐lineage cells in the periosteum.

## 2 Results

### 2.1 Mechanical Loading Induces Periosteal Bone Formation

**Figure 1:**
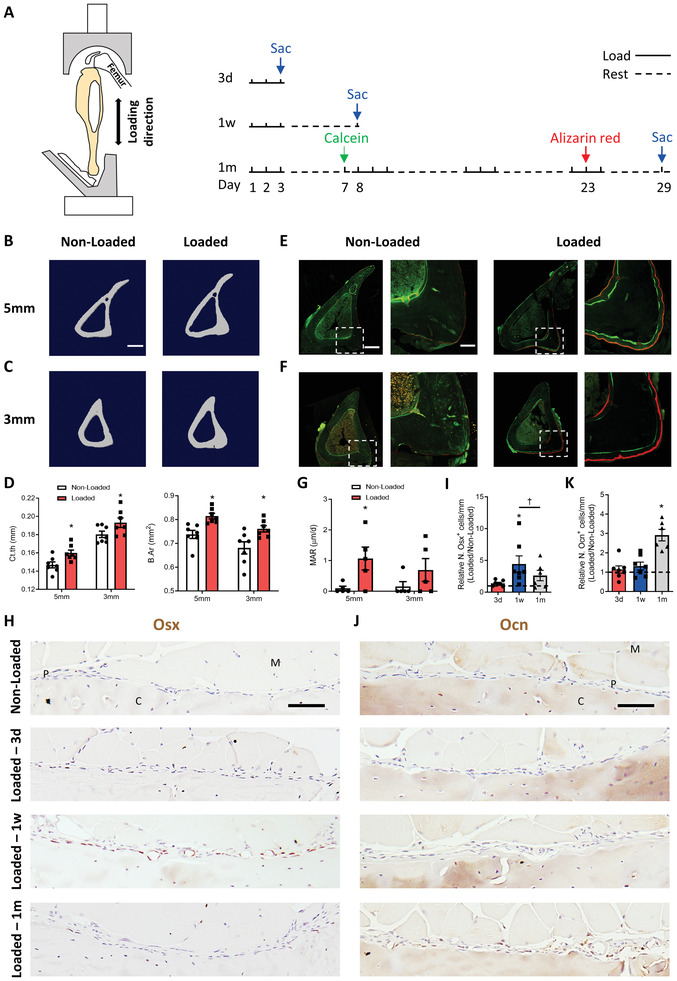
Axial compression loading induced periosteal bone formation. A) Loading regimen with calcein (green arrow) and alizarin red (red arrow) injections. Blue arrows indicate day of euthanasia. B–G) Wild type (WT) mice underwent one month of axial compression loading of tibiae, with calcein and alizarin red injected on days 6 and 22 after the first loading bout. Non‐loaded tibiae were used as controls. B,C) Representative microcomputed tomography (µCT) images and D) quantitative analysis of cortical thickness (Ct.th) and bone area (B.Ar) of tibial cortical bone at (B) 5 or (C) 3 mm proximal to the distal tibiofibular junction (TFJ) in mice. Scale bar, 500 µm. E,F) Left panels: representative images of dynamic histomorphometry analysis of tibiae at (E) 5 mm or (F) 3 mm proximal to the distal TFJ. Right panels: higher magnification of the boxed area in the left panels. Scale bar, 300 µm (left panels), 75 µm (right panels). G) Quantitative analysis of mineral apposition rate of the posterior and lateral surfaces of tibiae. H–K) Mice underwent 3 d, one week, or one month of axial compression loading of the tibiae. Non‐loaded tibiae were used as controls. H,J) Immunohistochemical staining and I,K) quantification of Osx^+^ cells (brown) and Ocn^+^ cells (brown) on the periosteal bone surface. Loaded tibiae values were normalized to the corresponding non‐loaded tibiae values. Scale bar, 50 µm. C, cortical bone; P, periosteum; M, muscle. 3d, 3 days; 1w, one week; 1m, one month. The analyses of (E–G, H–K) were performed at the same region of tibiae. Data are presented as mean ± standard error of the mean (SEM). *n* = 7 mice (D), *n* = 5 mice (G), *n* = 7 mice in 3 d or one week group and *n* = 6 mice in one month group (I, K). *p < 0.05 compared with the corresponding non‐loaded tibia; †*p* < 0.05. D,G) Statistical significance was determined by paired, two‐tailed *t*‐test. I,K) Statistical significance was determined by two‐way repeated measures analysis of variance (ANOVA) with Bonferroni post hoc test.

To examine mechanical loading‐induced lamellar periosteal bone formation, we performed an in vivo periodic axial compressive loading on tibiae of C57BL/6J mice 360 cycles per day at 2 Hz for 3 days (d), one week, or one month (**Figure** **1**A). The stress and strain distributions in the tibiae caused by compression were simulated in the finite element model. The measurements of von Mises stress and axial strain (E33) indicated that the stress concentration (Figure S1A,B, Supporting Information) and higher strain (Figure S1C,D, Supporting Information) were present at the mid‐diaphysis of mouse tibiae from 5 mm till 3 mm proximal to the distal tibiofibular junction (TFJ). Using microcomputed tomography (µCT), anabolic bone formation was observed in mice that underwent mechanical loading for one month (Figure 1B,C). Cortical thickness (Ct.Th) and bone area (B.Ar) were significantly greater in the loaded bone group than in the control group (Figure 1D). Calcein and alizarin were administered on days 6 and 22 after the first bout of loading to evaluate dynamic bone formation (Figure 1A). Consistent with µCT analysis, the loaded tibiae showed substantial osteogenesis and new bone formation, evident at the site of peak compressive strain on the periosteal surface (Figure 1E–G; Figure S1, Supporting Information). Mechanical loading produced significant anabolic responses at both 5 and 3 mm proximal to the distal TFJ. The site of 5 mm was selected as the representative anabolic region for the morphometric and histological analysis.

Histological analysis was conducted on sections of tibiae to examine cellular osteogenic responses to mechanical loading. Immunostaining of the site in the anabolic region which was at 5 mm proximal to the distal TFJ showed a significant increase in the number of osterix^+^ (Osx^+^) osteoprogenitors on the periosteal surface of the loaded tibiae after one week of loading, with no significant differences after 3 d and one month of loading. In addition, one week of loading induced a significantly greater number of periosteal Osx^+^ cells relative to one month of loading (Figure 1H,I). However, no significant elevation in the number of Osx^+^ cells was found after one week of loading at 7 or 1 mm proximal to the distal TFJ (Figure S2, Supporting Information). The observation indicates that the significant increase in the presence of Osx^+^ cells was at the site of peak strain, where the most bone growth occurred. The number of osteocalcin^+^ (Ocn^+^) cells was significantly greater on the periosteal surface of loaded tibiae in the anabolic region after one month of loading compared with the non‐loaded side, and no changes were observed after 3 d or one week of loading (Figure 1J,K). These results indicate that compression loading generates robust bone formation, especially on the periosteal bone surface. Furthermore, the periosteum may be highly sensitive to mechanical stimuli and creates an osteogenic microenvironment even after a short period of loading.

### 2.2 Increase of Myeloid‐Lineage Cells Is Correlated with TGF‐*β*1 Activation in the Periosteum in Response to Mechanical Loading

**Figure 2:**
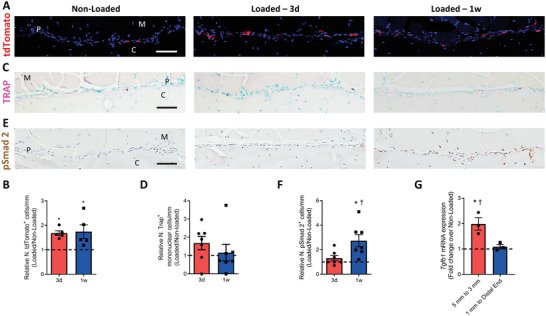
Myeloid‐lineage cells and transforming growth factor‐*β* 1 (TGF‐*β*1) activity were increased in the periosteum with mechanical loading. A–F) Mice underwent 3 d or one week of axial compression loading of the tibiae. Non‐loaded tibiae were used as controls. A) Immunofluorescent staining and B) quantification of tdTomato^+^ (red) cells on the periosteal tibial surface in *LysM‐cre::Ai14* mice. Blue indicates 4′,6‐diamidino‐2‐phenylindole (DAPI) staining of nuclei. Scale bar, 50 µm. C) Tartrate‐resistant acid phosphatase (TRAP) (magenta) staining and D) quantification on periosteal tibial surface in WT mice. Scale bar, 50 µm. E) Immunohistochemical staining and F) quantification of pSmad2^+^ cells (brown) on the periosteal tibial surface in WT mice. Scale bar, 50 µm. C, cortical bone; P, periosteum; M, muscle. 3d, 3 days; 1w, one week. G) WT mice underwent one week of axial compression loading of the tibiae. Non‐loaded tibiae were used as controls. mRNA expression of *Tgfb1* by reverse transcription quantitative polymerase chain reaction (RT‐qPCR) extracted from tibial cortical bone. The analyses of (A–F) were performed on the cross sections of posterior and lateral surfaces of tibiae at 5 mm proximal to the distal TFJ. Loaded tibiae values were normalized to the corresponding non‐loaded tibiae values. Data are presented as mean ± SEM. *n* = 5 mice at different time points (B) *n* = 7 at different time points (D, F) and *n* = 3 mice (G). B,D,F) *p < 0.05 compared with the corresponding non‐loaded tibia; †*p* < 0.05 compared with loaded tibiae of mice who underwent 3 d of loading. G) *p < 0.05 compared with the corresponding non‐loaded tibia. †*p* < 0.05 compared with the segment of loaded tibia which was from 1 mm proximal to the distal TFJ till the distal end. Statistical significance was determined by two‐way repeated measures ANOVA with Bonferroni post hoc test.

We have recently found that the deficiency in TRAP^+^ cells decreases cortical bone thickness,^[^ ^10^, ^27^ ^]^ which indicates the effect of myeloid lineage cells on periosteal bone formation. To examine the potential role of MCs in mechanical loading‐induced periosteal bone formation, we first analyzed the functional response of MCs to mechanical loading by using *LysM‐cre::Ai14* (*tdTomato*) mice. Interestingly, the number of periosteal tdTomato^+^ MCs in the anabolic region was significantly increased after 3 d of loading and remained elevated after one week of loading (**Figure** **2**A,B). However, the number of TRAP^+^ cells was only observed an increasing trend after 3 d of loading and no significant change after 3 d or one week of loading (Figure 2C,D). Co‐immunostaining of tdTomato and TRAP showed that mechanical loading did not alter the number of tdTomato^+^TRAP^+^ cells on the periosteal bone surface (Figure S3, Supporting Information). Thus, mechanical loading induces the increase of LysM^+^ but not TRAP^+^ periosteal MCs.

To further investigate the potential mechanism by which MCs regulate bone adaptation, we examined the activity of TGF‐*β* in the periosteum after mechanical loading, because TGF‐*β* is known to recruit mesenchymal stromal/stem cells for bone regeneration, modeling, and remodeling.^[^ ^20a^ ^]^ Immunostaining for phosphorylated Smad2 (pSmad2) revealed that the number of pSmad2^+^ cells increased significantly after one week of loading, but not after 3 d of loading at the site of 5 mm proximal to the distal TFJ (Figure 2E,F), similar to the temporospatial pattern of elevated Osx^+^ cells (Figure 1H,I). Few pSmad2^+^ cells were observed on the periosteal surface in the non‐loaded tibiae. These results suggest that active TGF‐*β*1 recruits Osx^+^ cells for the subsequential periosteal bone formation. The quantification of mRNA extracted from tibia showed *Tgfb1* expression was significantly upregulated by one week of loading in the segment from 5 mm till 3 mm proximal to the distal TFJ. Such effect was not observed in the segment from 1 mm proximal to the distal TFJ till the end of the tibia (Figure 2G). Moreover, there was no significant change in the numbers of tdTomato^+^ (Figure S4A,B, Supporting Information) and pSmad2^+^ cells (Figure S4C,D, Supporting Information) between loaded and non‐loaded tibiae at the site of 7 or 1 mm proximal to the distal TFJ. Collectively, our results reveal that TGF‐*β* activity increases in response to mechanical loading, and this increase has a temporospatial correlation with higher numbers of LysM^+^TRAP^−^ MCs on the periosteal bone surface.

### 2.3 TGF‐*β*1 Secreted from Periosteal Myeloid‐Lineage Cells Triggers Mechanical Loading‐Induced Periosteal Bone Formation

**Figure 3:**
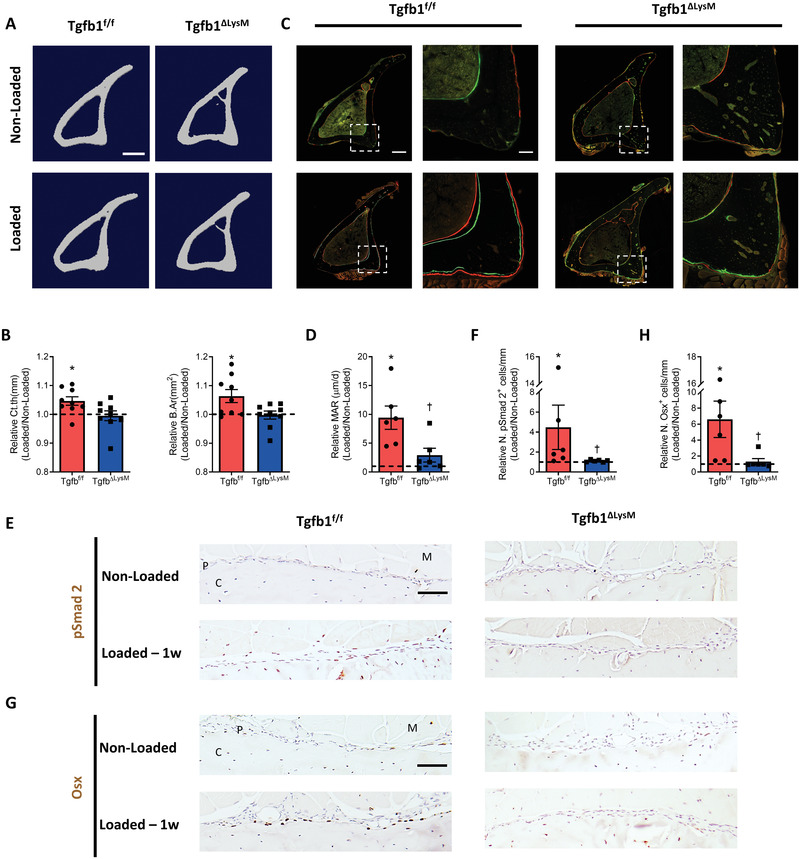
Knockout of *Tgfb1* in the LysM^+^ myeloid‐lineage cells attenuated loading‐induced periosteal bone formation. A–D) *Tgfb1^flox/flox^* or *Tgfb1^ΔLysM^* mice underwent one month of axial compression loading of tibiae, with calcein and alizarin red injected on days 6 and 22 after the first loading bout. Non‐loaded tibiae were used as controls. A) Representative µCT images and B) quantitative analysis of cortical thickness (Ct.th) and bone area (B.Ar) of tibial cortical bone at 5 mm proximal to the distal TFJ in mice. Scale bar, 500 µm. C) Left panels: representative images of dynamic histomorphometry of tibiae at 5 mm proximal to the distal TFJ. Right panels: higher magnification of the boxed area in the left panels. Scale bar, 300 µm (left panels), 75 µm (right panels). D) Quantitative analysis of mineral apposition rate of the posterior and lateral surfaces of tibiae. E–H) Mice underwent one week of axial compression loading of tibiae. Non‐loaded tibiae were used as controls. E,G) Immunohistochemical staining and F,H) quantification of pSmad2^+^ cells (brown) and Osx^+^ cells (brown) on the periosteal bone surface. Scale bar, 50 µm. C, cortical bone; P, periosteum; M, muscle. 1w, one week. The analyses of (C, D, E–H) were performed at the same region of tibiae. Loaded tibiae values were normalized to the corresponding non‐loaded tibiae values. Data are presented as mean ± SEM. *n* = 9 mice per group (B) and *n* = 6 mice per group (D, F, H). *p < 0.05 compared with the corresponding non‐loaded tibia; †*p* < 0.05 compared with loaded tibiae of *Tgfb1^flox/flox^* mice. Statistical significance was determined by two‐way repeated measures ANOVA with Bonferroni post hoc test.

MCs are known to secrete TGF‐*β*1 for tissue homeostasis and regeneration.^[^ ^18^, ^19^ ^]^ To investigate whether periosteal MCs mediate TGF‐*β* activities in response to mechanical loading, *Tgfb1^flox/flox^* ^^mice were crossed with *LysM‐cre* mice to generate *LysM‐cre::Tgfb1^flox/flox^* ^^(*Tgfb1* ^ΔLysM^) mice, in which LysM^+^ MCs no longer express TGF‐*β*1. Cortical bone formation was significantly increased in *Tgfb1^flox/flox^* mice after one month of mechanical loading, including the increases of Ct.th and B.Ar in µCT analysis, whereas the anabolic responses to mechanical loading were diminished with deletion of *TGF‐β1* from LysM^+^ MCs in *Tgfb1* ^ΔLysM^ mice (**Figure** **3**A,B). Dynamic histomorphometry also showed reduced periosteal bone mineral apposition rate (Figure 3C,D) in *Tgfb1* ^ΔLysM^ mice relative their WT littermates (*Tgfb1^flox/flox^* ^^mice). Furthermore, the increase in the number of pSmad2^+^ cells after loading on the periosteal bone surface was inhibited in *Tgfb1* ^ΔLysM^ mice, as illustrated in immunostained sections (Figure 3E,F). Importantly, the number of Osx^+^ (Figure 3G,H) cells on the periosteal bone surface progressively increased after loading in *Tgfb1^flox/flox^* mice but not in *Tgfb1* ^ΔLysM^ mice. These data demonstrate that TGF‐*β*1 secreted by LysM^+^ MCs is essential to initiate mechanical loading‐induced periosteal bone formation.

### 2.4 Mechanical Loading Promotes Accumulation of Periosteal Myeloid‐Lineage Cells

**Figure 4:**
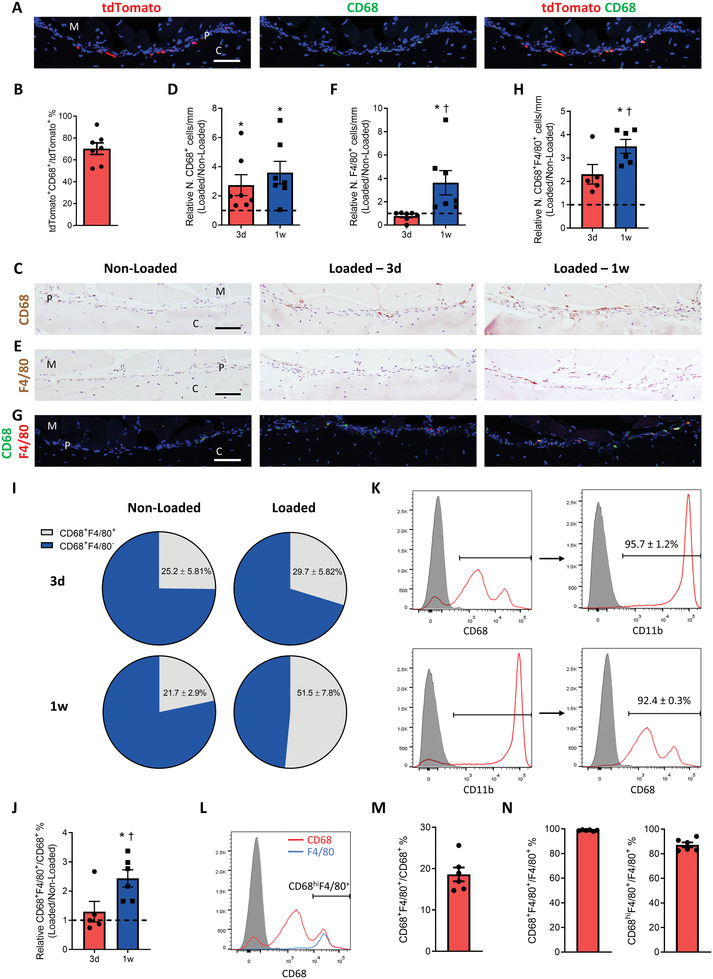
Mechanical loading increased periosteal cluster of differentiation (CD)68^+^F4/80^+^ macrophages. A) Immunofluorescent staining and B) quantification of tdTomato^+^ (red) and CD68^+^ (green) cells on the periosteal tibial surface in *LysM‐cre::Ai14* mice. Blue indicates DAPI staining of nuclei. Scale bar, 50 µm. C–I) WT mice underwent 3 d or one week of axial compression loading of tibiae. Non‐loaded tibiae were used as controls. C,E) Immunohistochemical staining and D,F) quantification of CD68^+^ (brown) and F4/80^+^ cells (brown) on the periosteal tibial surface. Scale bar, 50 µm. G) Immunofluorescent staining and H) quantification of CD68^+^ (green) and F4/80^+^ (red) cells on the periosteal tibial surface. Blue indicates DAPI staining of nuclei. Scale bar, 50 µm. C, cortical bone; P, periosteum; M, muscle. I,J) The percentage of CD68^+^ cells that express F4/80. 3d, 3 days; 1w, one week. The analyses of (A–I) were performed on the cross‐sectional sections of the posterior and lateral surfaces of tibiae at 5 mm proximal to the distal TFJ. Loaded tibiae values were normalized to the corresponding non‐loaded tibiae values. K,L) The flow cytometry analysis of CD11b^+^, CD68^+^, and F4/80^+^ cells in the bone marrow and periosteum of WT mice. K) Upper panels: the percentage of CD68^+^ cells that express CD11b. Lower panels: the percentage of CD11b^+^ cells that express CD68. The gray shaded area shows isotype control and the red line shows anti‐CD11b or anti‐CD68 antibody stain. L) Representative images of flow cytometry analysis, M) the percentage of the CD68^+^ cells that express F4/80 and N) the percentage of F4/80^+^ cells that express CD68 or high level of CD68 (CD68^hi^). The gray shaded area shows istotype control, the red line shows anti‐CD68 antibody stain, and the blue line shows anti‐F4/80 antibody stain. Data are presented as mean ± SEM. *n* = 7 mice per group (B, D, F), *n* = 5 mice in 3 d group and *n* = 6 mice in one week group (H–J), *n* = 6 mice (K–N). D,F,H,J) *p < 0.05 compared with the corresponding non‐loaded tibia; †*p* < 0.05 compared with loaded tibiae of mice who underwent 3 d of loading. Statistical significance was determined by two‐way repeated measures ANOVA with Bonferroni post hoc test.

We next attempted to characterize the subtypes of periosteal LysM^+^ MCs. Macrophages are essential in periosteal bone formation and regeneration.^[^ ^10^, ^27^ ^]^ The expressions of CD68 and F4/80 were then examined in the periosteum of limb bones using *LysM‐cre::tdTomato* and WT mice because CD68 and F4/80 antigens are robust pan‐macrophage markers in mice.^[^ ^28^ ^]^ Immunostaining of tibiae sections showed that approximately 67% of LysM^+^ cells were CD68^+^ (**Figure** **4**A,B) and 92% of CD68^+^ were LysM^+^ (Figure S5A, Supporting Information) in the periosteum, indicating that CD68^+^ cells are the predominant subtype of periosteal LysM^+^ MCs. We then examined the temporospatial changes of periosteal CD68^+^ MCs on the tibiae during anabolic bone formation in response to mechanical loading. Consistent with the observations of LysM^+^ MCs (Figure 2A,B), the number of periosteal CD68^+^ MCs were significantly elevated after 3 days and 1 week of loading in WT mice (Figure 4C,D). However, the number of F4/80^+^ cells did not change after 3 d of loading but were significantly increased after one week (Figure 4E,F). Co‐immunostaining of CD68 and F4/80 revealed that approximately 23% of CD68^+^ MCs expressed F4/80 (Figure 4G), but most F4/80^+^ periosteal macrophages expressed CD68 (Figure S5B, Supporting Information). Furthermore, the increase of CD68^+^F4/80^+^ macrophages was detected after one week of loading but not after 3 d (Figure 4G,H), and the similar temporospatial pattern of pSmad2^+^ cells was observed on the periosteal bone surface (Figure 2E,F). Additionally, one week of loading induced a significantly higher percentage of periosteal CD68^+^ cells that expressed F4/80^+^, whereas 3 d of loading did not (Figure 4I,J). Instead, no significant changes in the number of CD68^−^F4/80^+^ cells were observed after mechanical loading (Figure S6, Supporting Information), suggesting that CD68^−^F4/80^+^ cells were not responsible for loading‐induced bone formation. Moreover, CD68^+^ macrophages in bone marrow and periosteum were analyzed using flow cytometry. Nearly all CD68^+^ cells were positive for myeloid marker CD11b, and almost all CD11b^+^ cells were positive for CD68 (Figure 4K). Interestingly, we also observed two distinct levels of CD68 expression in CD68^+^ cells: low‐level expression (CD68^lo^) and high‐level expression (CD68^hi^). F4/80^+^ cells were mostly expressed in the CD68^hi^ subtype (CD68^hi^F4/80^+^) (Figure 4L–N), consistent with our observation of immunostaining of the periosteal bone surface. Collectively, mechanical loading induces the increase of periosteal F4/80^+^ macrophages, the subtype of CD68^+^ MCs, suggesting that CD68^+^F4/80^+^ macrophages may be differentiated from CD68^+^F4/80^−^ MCs in the periosteum in response to mechanical loading.

### 2.5 CD68^+^F4/80^+^ Macrophage Is the Primary Subtype Expressing TGF‐*β*1

**Figure 5:**
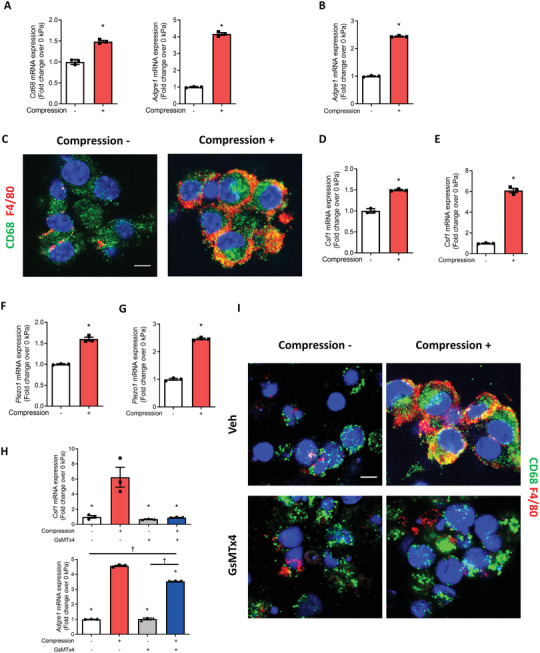
Mechanical compression stimulated piezo‐type mechanosensitive ion channel component 1 (PIEZO1) signaling for CD68^+^F4/80^+^ macrophages differentiation in whole periosteal cells (WPCs) and bone marrow‐derived macrophages (BMDMs). A–I) Primary WPCs or BMDMs were harvested from WT mice and subjected to 10 or 0 kPa of mechanical compression. A) mRNA expression of *Cd68* and adhesion G protein‐coupled receptor E1 (*Adgre1*) by RT‐qPCR in WPCs after compression. B) mRNA expression of *Adgre1* in BMDMs after compression. C) Immunofluorescent staining of CD68^+^ (green) and F4/80^+^ (red) cells in BMDMs after compression. Blue indicates DAPI staining of nuclei. Scale bar, 5 µm. D,E) mRNA expression of colony‐stimulating factor 1 (*Csf1*) in WPCs and BMDMs after compression, respectively. F,G) mRNA expression of *Piezo1* in WPCs and BMDMs after compression, respectively. H,I) BMDMs were pretreated with the PIEZO1 inhibitor GsMTx4 or vehicle for 24 h before compression. H) mRNA expression of *Csf1* and *Adgre1* in BMDMs after compression. I) Immunofluorescent staining of CD68^+^ (green) and F4/80^+^ (red) cells after compression. Blue indicates DAPI staining of nuclei. Scale bar, 5 µm. Data are presented as mean ± SEM. *n* = 3 per group. A,B,D–G) * *p* < 0.05 compared with 0 kPa. Statistical significance was determined by unpaired, two‐tailed Student's *t*‐test. H) *p < 0.05 compared with vehicle 10‐kPa compression group; †*p* < 0.05. Statistical significance was determined by two‐way ANOVA with Bonferroni post hoc test.

To identify potential factors that induce CD68^+^ MC differentiation in response to mechanical loading, we isolated primary whole periosteal cells (WPCs) from WT mice and applied compression loading using the Flexcell Compression System. Consistent with our in vivo results, expression of both *CD68* and F4/80‐coding gene adhesion G protein‐coupled receptor E1 (*Adgre1*) in the WPCs were significantly increased after 3 days of compression loading, as indexed by mRNA levels (**Figure** **5**A). The primary bone marrow‐derived macrophages (BMDMs) were also examined in the Flexcell system with compression loading. The optimal compression condition of 10 kPa for 4 hours (h) was determined by viability staining (Figure S7, Supporting Information). Similarly, *Adgre1* expression in BMDMs was also significantly increased after loading (Figure 5B). Immunostaining for CD68 and F4/80 showed that compression loading induced F4/80 expression on most CD68^+^ cells (Figure 5C). Colony‐stimulating factor 1 (Csf1) is essential for survival, differentiation, and proliferation of MCs. It is secreted primarily by mesenchymal but also produced by macrophages.^[^ ^29^ ^]^ Interestingly, compression loading also induced upregulation of *Csf1* expression in WPCs and BMDMs (Figure 5D,E). We then sought to determine the mechanosensation of CD68^+^ MCs. Myeloid‐lineage cells sense mechanical fluctuations and generate an immune response in the lung.^[^ ^16^ ^]^ Piezo1, a mechanosensitive ion channel, is directly activated by forces exerted on cell membranes. Our results showed that compression loading significantly upregulated the expression of *Piezo1* in WPCs and BMDMs (Figure 5F,G). Importantly, the expression of *Csf1* and *Adgre1* induced by compression loading was diminished by Piezo1 inhibitor GsMTx4 (Figure 5H). In addition, the inhibition of Piezo1 resulted in the loss of F4/80 expression in CD68^+^ cells after compression (Figure 5I). These results suggest that Piezo1 signaling mediates CD68^+^F4/80^−^ MC differentiation to CD68^+^F4/80^+^ macrophages with synthesis of Csf1 in response to mechanical loading.

**Figure 6:**
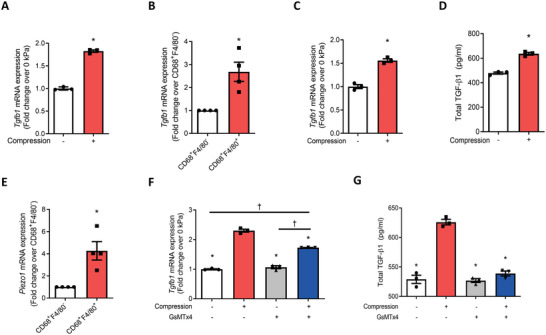
CD68^+^F4/80^+^ macrophages induced secretion of TGF‐*β*1 via PIEZO1 signaling in response to mechanical compression. A) mRNA expression of *Tgfb1* in primary WPCs harvested from WT mice and treated with 10 or 0‐kPa mechanical compression for 3 d. B) mRNA expression of *Tgfb1* in CD68^+^F4/80^+^ and CD68^+^F4/80^−^ cells sorted from *hCD68‐GFP* mice. C,D) Primary BMDMs were harvested from WT mice and subjected to 10 or 0‐kPa mechanical compression. C) mRNA expression of *Tgfb1* in BMDMs after 4 h of compression. D) Total TGF‐*β*1 in the conditioned medium of the BMDMs culture after 4 h of compression and an additional 12 h of uncompressed incubation determined by enzyme‐linked immunosorbent assay (ELISA). E) mRNA expression of *Piezo1* in CD68^+^F4/80^+^ and CD68^+^F4/80^−^ cells sorted from *hCD68‐GFP* mice. F,G) BMDMs were pretreated with the PIEZO1 inhibitor GsMTx4 or vehicle for 24 h followed by 10 or 0 kPa of mechanical compression. F) mRNA expression of *Tgfb1* in BMDMs after 4 h of compression. G) Total TGF‐*β*1 in the conditioned medium of the BMDMs culture after 4 h of compression and an additional 12 h of uncompressed incubation determined by ELISA. Data are presented as mean ± SEM. *n = 3* (A, C, D, F, G) and *n* = 4 mice (B, E) A, C, D) *p < 0.05 compared with 0 kPa. B, E) * *p < 0.05* compared with CD68^+^F4/80^‐^ cells. Statistical significance was determined by unpaired, two‐tailed Student's *t*‐test. F,G) *p < 0.05 compared with vehicle 10‐kPa compression group; †*p* < 0.05. Statistical significance was determined by two‐way ANOVA with Bonferroni post hoc test.

We next investigated whether CD68^+^F4/80^+^ macrophages regulate TGF‐*β*1 activities in response to mechanical loading. Significant upregulation of *Tgfb1* expression in the WPCs was observed in 10‐kPa compression loading relative to 0‐kPa loading (**Figure** **6**A). CD68, as an intracellular marker, requires fixation and permeabilization before fluorescence‐activated cell sorting. Therefore, live CD68^+^F4/80^−^ and CD68^+^F4/80^+^ cells were sorted from bone marrow and periosteum of *hCD68‐GFP* transgenic mice. The expression of *Tgfb1* in CD68^+^F4/80^+^ cells was significantly higher relative to CD68^+^F4/80^−^ cells (Figure 6B). Additionally, *Tgfb1* mRNA expression in primary BMDMs was also significantly increased with compression loading (Figure 6C). The levels of TGF‐*β*1 protein were measured in the conditioned medium from BMDM culture subjected with or without compression loading by enzyme‐linked immunosorbent assay (ELISA). Mechanical loading stimulated the secretion of TGF‐*β*1 (Figure 6D). Interestingly, the expression of *Piezo1* was also approximately 4 times higher in CD68^+^F4/80^+^ cells relative to CD68^+^F4/80^−^ cells (Figure 6E). As expected, GsMTx4 inhibited mechanical loading‐induced upregulation of *Tgfb1* in BMDMs (Figure 6F) and TGF‐*β*1 protein level in the conditioned medium of BMDMs by ELISA (Figure 6G). Together, Piezo1 signaling induces differentiation of CD68^+^F4/80^−^ MCs into the CD68^+^F4/80^+^ macrophage subtype, which secretes TGF‐*β*1 in response to mechanical stimulation.

### 2.6 CD68^+^F4/80^+^ Macrophages Mediate Mechanical Loading‐Induced TGF‐*β*1 Activation

**Figure 7:**
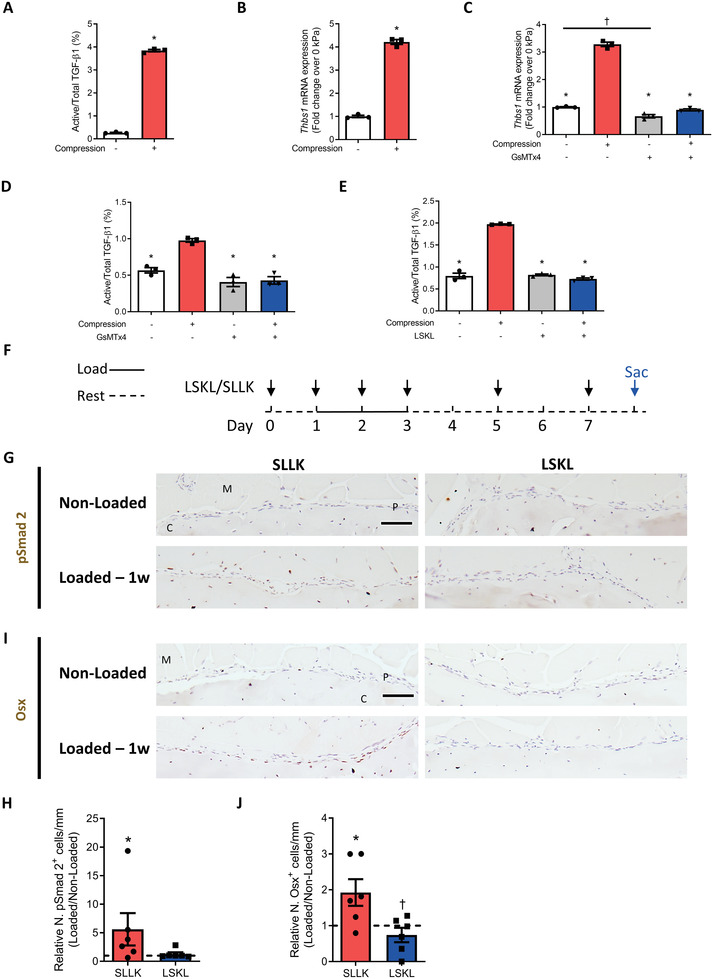
CD68^+^F4/80^+^ macrophages activated TGF‐*β*1 by secretion of Thbs1 in response to mechanical loading. A–E) Primary BMDMs were harvested from WT mice and subjected to 10 or 0‐kPa mechanical compression. A) Active TGF‐*β*1 in the conditioned medium of the BMDM culture after 4 h of compression and an additional 12 h of uncompressed incubation determined by ELISA. B) mRNA expression of thrombospondin‐1 (*Thbs1*) in BMDMs after 4 h of compression. C,D) BMDMs were pretreated with the PIEZO1 inhibitor GsMTx4 or vehicle for 24 h before compression. C) mRNA expression of *Tgfb1* in BMDMs after 4 h of compression. D) TGF‐*β*1 in the conditioned medium of the BMDM culture after 4 h of compression and an additional 12 h of uncompressed incubation determined by ELISA. E) BMDMs were pretreated with the Thbs1 inhibitor leucine‐serine‐lysine‐leucine (LSKL) or control peptide serine‐leucine‐leucine‐lysine (SLLK) for 24 h before compression. TGF‐*β*1 in the conditioned medium of the BMDM culture after 4 h compression and additional 12 h uncompressed incubation determined by ELISA. F–J) WT mice underwent one week of axial compression loading of tibiae, with LSKL or SLLK injections. Non‐loaded tibiae were used as controls. F) Treatment regimen with LSKL or SLLK (black arrows). Blue arrows indicate the day of euthanasia. G,I) Immunohistochemical staining and H,J) quantification of pSmad2^+^ cells (brown) and Osx^+^ cells (brown) on the periosteal bone surface. Scale bar, 50 µm. C, cortical bone; P, periosteum; M, muscle. 1w, 1 week. The analyses of (G–J) were performed on the cross‐sectional sections of the posterior and lateral surfaces of the tibiae at 5 mm proximal to the distal TFJ. Loaded tibiae values were normalized to the corresponding non‐loaded tibiae values. Data are presented as mean ± SEM. *n* = 3 per group (A–E) and *n* = 6 mice per group (H, J). A,B) *p < 0.05 compared with 0 kPa. Statistical significance was determined by unpaired, two‐tailed Student's *t*‐test. C–E) *p < 0.05 compared with vehicle 10‐kPa compression group; †*p* < 0.05. Statistical significance was determined by two‐way ANOVA with Bonferroni post hoc test. H,J) *p < 0.05 compared with the corresponding non‐loaded tibia; †*p* < 0.05 compared with loaded tibiae of mice who were treated with SLLK. Statistical significance was determined by two‐way repeated measures ANOVA with Bonferroni post hoc test.

TGF‐*β*1 is synthesized and secreted in an inactive latent form. The disruption of the interaction between active TGF‐*β* and LAP is required for TGF‐*β* to be biologically active. We finally investigated the mechanism of TGF‐*β*1 activation by CD68^+^F4/80^+^ macrophages with mechanical loading. The levels of active TGF‐*β*1 were four times higher in the conditioned medium of BMDMs culture with 10‐kPa compression loading relative to 0‐kPa loading (**Figure** **7**A). Macrophages have been shown to activate latent TGF‐*β*1 by secretion of Thbs1.^[^ ^24^ ^]^ Notably, *Thbs1* expression was significantly upregulated with compression loading in reverse transcription‐quantitative polymerase chain reaction (RT‐qPCR) (Figure 7B) and Western blot analysis (Figure S8, Supporting Information). With the addition of GsMTx4 treatment, increases of *Thbs1* expression in BMDMs and active TGF‐*β*1 released in the conditioned medium induced by compression loading were completely inhibited (Figure 7C,D). Moreover, the LSKL peptide, an inhibitor of Thbs1, abolished the effect of compression loading on TGF‐*β*1 activation (Figure 7E). We further validated Thbs1 in activation of TGF‐*β*1 in the tibial compression mouse model. LSKL was administered to WT mice before one week of mechanical loading (Figure 7F). As expected, compression loading activated TGF‐*β*1, as evidenced by the increased number of pSmad2^+^ cells on the periosteal bone surface in the control peptide group. Importantly, LSKL significantly decreased the number of pSmad2^+^ cells on the periosteal bone surface (Figure 7G,H), consistent with the results of the BMDM in vitro experiment. Furthermore, the increase of Osx^+^ osteoprogenitors by mechanical loading was suppressed by LSKL administration (Figure 7I,J). Together, these results show that CD68^+^F4/80^+^ macrophages not only secrete TGF‐*β*1 in response to mechanical loading but also activate latent TGF‐*β*1 via Thbs1 signaling.

## 3 Discussion

Bone mass and microarchitecture are regulated by mechanical loading.^[^ ^30^ ^]^ Osteocytes are believed to be the main mechanosensor to orchestrate loading‐induced bone adaptation.^[^ ^31^ ^]^ Previous studies of in vivo osteocyte function have focused predominantly on trabecular bone remodeling.^[^ ^31^, ^32^ ^]^ Our data and other studies^[^ ^8^ ^]^ have shown that mechanical loading induces significant periosteal bone formation. However, the mechanisms of periosteal expansion of cortical bones in response to mechanical loading remain poorly understood. Our findings provide direct evidence that periosteal myeloid‐lineage cells potentiate the anabolic response of cortical bone to mechanical stimuli.

**Figure 8:**
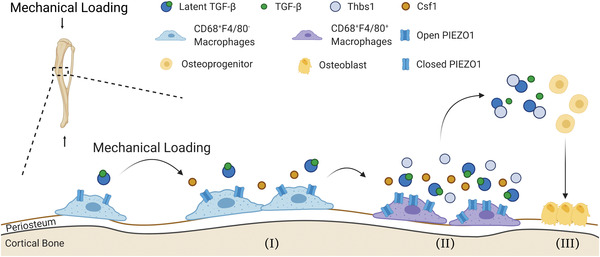
Schematic of macrophages in regulation of mechanical loading‐induced periosteal bone formation. Under mechanical stimuli, (I) periosteal CD68^+^F4/80^−^ myeloid‐lineage cells are increased and (II) differentiate into CD68^+^F4/80^+^ macrophages with the synthesis of Csf1 via Piezo1 signaling. CD68^+^F4/80^+^ macrophages secrete TGF‐*β*1 and further activate TGF‐*β*1 by producing Thbs1. (III) Active TGF‐*β*1 recruits osteoprogenitors to the periosteal bone surface for bone formation.

Periosteum provides an osteogenic microenvironment with progenitors, nerves, vasculature, matrix proteins for cortical bone growth and modeling. Loss of periosteal progenitors^[^ ^33^ ^]^ or periostin^[^ ^34^ ^]^ diminishes loading‐induced periosteal bone formation. These observations suggest that periosteum has a critical role in strain‐adaptive cortical bone modeling. Moreover, tropomyosin receptor kinase A‐positive (TrkA^+^) sensory nerves^[^ ^8c^ ^]^ and Piezo1 signaling in osteoblast‐lineage cells^[^ ^8a^ ^]^ contribute to periosteal bone formation in response to mechanical loading. Interestingly, the deficiency of TrkA^+^ nerves or the deletion of Piezo1 in osteocytes still significantly increased cortical bone formation after mechanical loading.^[^ ^8a,c^ ^]^ Thus, other cellular and molecular mechanisms may participate in loading‐induced osteoanabolic response in the periosteum. Our previous study showed that periosteal macrophage‐lineage TRAP^+^ mononuclear cells are essential in maintaining periosteum homeostasis and periosteal bone formation.^[^ ^10c^ ^]^ Surprisingly, the current study showed that the number of these cells did not increase significantly with mechanical loading (Figure 2C,D). It indicated that TRAP^+^ mononuclear cells were unlikely to facilitate mechanical loading‐induced bone formation. Indeed, we revealed that another subtype of periosteal macrophages regulated cortical bone modeling engendered by mechanical loading. Mechanical stress‐induced Piezo1 expression and signaling promoted the differentiation of periosteal CD68^+^F4/80^+^ macrophages from CD68^+^F4/80^−^ MCs. Importantly, periosteal CD68^+^F4/80^+^ macrophages secreted and activated TGF‐*β*1 for recruitment of osteoprogenitor cells to the periosteal bone surface, resulting in osteogenesis following mechanical loading (**Figure** **8**).

Macrophages are present in most organs and tissues to support homeostasis, development, and regeneration. We have reported that cortical bone formation was impaired in mice with *Csf1* deficiency, where macrophages were eliminated.^[^ ^10c^ ^]^ Consistent with our findings, cortical bone volume and density were diminished with the depletion of macrophages.^[^ ^12^, ^13^ ^]^ Osteal macrophages positive for CD68 and F4/80 have been characterized as non‐osteoclast macrophages that support osteoblast function.^[^ ^14a^ ^]^ They are located on the periosteal and endosteal surfaces of cortical bone, as well as in the bone marrow. Interestingly, we found CD68^+^F4/80^−^ MCs were first significantly increased in the mechanically stimulated periosteum, followed by the acquisition of F4/80 expression as a subtype of CD68^+^ MCs. Specifically, CD68^+^F4/80^−^ MCs differentiated into CD68^+^F4/80^+^ macrophages in response to mechanical loading. Our study demonstrates the role of periosteal MCs in loading‐induced cortical bone formation. Owing to the lack of transgenic mouse strain specifically labeling periosteal MCs, the effect of MCs residing in other tissues cannot be ruled out on the periosteal bone formation.

Macrophages from bones have been reported to secrete TGF‐*β*1 after efferocytosis of apoptotic osteoblasts in vitro.^[^ ^35^ ^]^ TGF‐*β*1 mobilizes and recruits mesenchymal stem cells for tissue repair and remodeling. We previously demonstrated that active TGF‐*β*1 is a coupling factor for osteoclastic bone resorption and osteoblastic bone formation during bone remodeling. It recruits mesenchymal progenitor cells to bone resorption sites for bone formation through pSmad2/3 signaling pathway.^[^ ^20a^ ^]^ Our data here showed that mechanical loading initiated the secretion and activation of TGF‐*β*1 by periosteal macrophages. Importantly, we identified CD68^+^F4/80^+^ macrophages as the main subtype of TGF‐*β*1 producing macrophages, as demonstrated by the elevated number of CD68^+^F4/80^+^ macrophages, and their increased expression and secretion of TGF‐*β*1 after mechanical loading. Moreover, ablation of *Tgfb1* in MCs caused a significant reduction in loading‐induced expression of periosteal osteoprogenitors and periosteal bone formation, further validating our findings. Secreted TGF‐*β*1 is in the inactive form and requires activation to function.^[^ ^36^ ^]^ Our study demonstrated that CD68^+^F4/80^+^ macrophages secreted Thbs1 to activate TGF‐*β*1 under mechanical stress. With the administration of Thbs1 inhibitor, the mechanical loading–induced TGF‐*β*1 activation and increase of periosteal osteoprogenitors were abolished. Therefore, under mechanical loading, periosteal macrophages produce and activate TGF‐*β*1 to recruit osteoprogenitors for periosteal bone formation.

Piezo1 has been proposed to mediate immune responses,^[^ ^16^ ^]^ bone remodeling,^[^ ^8^, ^37^ ^]^ and cell fate determination.^[^ ^38^ ^]^ Knockout of *Piezo1* in osteoblast lineage cells resulted in a low bone mass phenotype and alleviated bone loss caused by hindlimb suspension.^[^ ^37^ ^]^ However, hindlimb suspension induces significant adaptations in the cardiovascular system, nervous system, and immune system,^[^ ^39^ ^]^ which may directly or indirectly affect bone metabolic activities, confounding the contribution of Piezo1 in skeletal response. Further investigation is necessary to delineate the specific effect of Piezo1 on mechanical loading‐induced bone adaptation. Our study showed that the inhibition of Piezo1 channel diminished the differentiation of CD68^+^F4/80^−^ MCs into CD68^+^F4/80^+^ macrophages under compression loading. CD68^+^F4/80^+^ macrophages expressed higher levels of *Tgfb1* compared with CD68^+^F4/80^−^ MCs. In this regard, the mechanical loading‐induced secretion of TGF‐*β*1 is likely due to the increased number of CD68^+^F4/80^+^ macrophages. Alternatively, Piezo1 activation triggers calcium influx into the cytoplasm,^[^ ^16^, ^17^ ^]^ which may upregulate mitogen‐activated protein kinase or phosphoinositide 3‐kinase signaling for TGF‐*β*1 production.^[^ ^40^ ^]^ These results suggest that when artificial forces are applied directly to the tibia, periosteal MCs express Piezo1 to sense mechanical stimuli, and in turn, Piezo1 signaling promotes CD68^+^F4/80^+^ macrophages differentiation for cortical bone expansion. In summary, periosteal MCs undergo CD68^+^F4/80^+^ macrophages differentiation in response to mechanical loading to secret and activate TGF‐*β*1 for cortical bone formation. Our findings, to the best of knowledge, provide the first direct evidence that periosteal MCs orchestrate cortical bone formation under mechanical stimulation.

## 4 Experimental Section

### Mouse Models

C57BL/6J (WT, stock no. 000664), *LysM‐cre* (stock no. 004781), *Ai14* (stock no. 007914), *Tgfb1^flox/flox^* (stock no. 010721), and *hCD68‐GFP* (stock no. 026827) mouse strains were purchased from the Jackson Laboratory (Bar Harbor, ME). *LysM‐cre* mice were crossed with *Ai14* or *Tgfb1^flox/flox^*. The offspring were intercrossed to generate the following genotypes: WT, *LysM‐cre* (mice expressing Cre recombinase driven by *LysM* promoter), *LysM‐cre::Ai14* (mice express tdTomato fluorescence by *LysM* lineage cells, referred to as *LysM‐cre::tdTomato* herein), *Ai14, LysM‐cre::Tgfb1^flox/flox^* (conditional deletion of *Tgfb1* in *LysM* lineage cells, referred to as *Tgfb1* ^ΔLysM^ herein), and *Tgfb1^flox/flox^* (mice homozygous for the Tgfb1 floxed allele) mice. The genotypes of the mice were determined by PCR analyses of genomic DNA isolated from mouse tails with the following primers: *LysM‐cre*: common: 5′‐AAGGAGGGACTTGGAGGATG‐3′, WT reverse: 5′‐GTCACTCACTGCTCCCCTGT‐3′, mutant reverse: 5′‐ACCGGTAATGCAGGCAAAT‐3′; *Ai14*: WT forward: 5′‐AAGGGAGCTGCAGTGGAGTA‐3′, WT reverse: 5′‐CCGAAAATCTGTGGGAAGTC‐3′, mutant reverse: 5′‐GGCATTAAAGCAGCGTATCC‐3′, mutant forward: 5′‐CTGTTCCTGTACGGCATG G‐3′; *Tgfb1^flox/flox^*: forward: 5′‐AAGACCTGGGTTGGAAGTG‐3′, reverse: 5′‐CTTCTCCGTTTCTCTGTCACCCTA T‐3′; *hCD68‐GFP*: transgene forward: 5′‐CGGCTCTGTGAATGACAATG‐3′, transgene reverse: 5′‐TTGGCAGTTGTGGCAAGTAG‐3′, internal positive control forward: 5′‐CTAGGCCACAGAATTGAAAGATCT‐3′, internal positive control reverse: 5′‐ GTAGGTGGAAATTCTAGCATCATCC‐3′. All animals were maintained in the animal facility of The Johns Hopkins University School of Medicine (Baltimore, MD).

### Mechanical Loading

Mechanical loading of 16‐week‐old male mice was performed as previously described.^[^ ^41^ ^]^ Briefly, the mice were anesthetized with isoflurane (Forane, Baxter International Inc., Deerfield, IL) for the duration of the experiment. The left tibia was axially compressed by fixing the knee and ankle into molded cups on the electromagnetic mechanical actuator (ElectroForce 5500, TA Instruments, New Castle, DE). Loading was applied with a continuous 2‐Hz sinusoidal waveform ranging from 2‐N compressive loading and a 12‐N peak loading for 360 cycles per day on three consecutive days per week. The right tibia served as the internal control. Unrestricted cage activity was allowed between loading bouts. For the time‐course experiments, mice were euthanized after 3 d, one week, or one month of loading (*n* = 5–9 per group). Dynamic histomorphometry was performed on the mice in the one‐month loading group. Calcein (10 mg kg^−1^, C0857, Sigma‐Aldrich, St. Louis, MO) and alizarin (30 mg kg^−1^, A3882, Sigma‐Aldrich) were injected intraperitoneally into the mice on days 6 and 22 after the first loading bout. For the Thbs1 inhibitor treatment experiments, WT mice in the one‐week loading group were assigned randomly into two subgroups (*n* = 6 per subgroup). Thbs1 inhibitor (LSKL) and control peptide serine‐leucine‐leucine‐lysine (SLLK) were synthesized with purity >98% (Biomatik USA LLC, Wilmington, DE). One day before the first loading bout, LSKL (30 mg kg^−1^) or the equivalent volume of SLLK was administered intraperitoneally (one injection) during the 3‐d loading session (three injections in total), and one injection every other day during the 4‐d resting session (two injections in total).

### Cell Sorting and Flow Cytometry Analysis

Cell sorting and flow cytometry analysis of CD68^+^ cells were performed from *hCD68‐GFP* and WT mice, respectively. Hindlimbs were collected with intact periosteum after the surrounding muscle tissues were carefully removed. Bone marrow cells were flushed with a fluorescence‐activated cell sorting (FACS) buffer (5% fetal bovine serum [FBS] in phosphate‐buffered saline [PBS]), and the bones were crushed with sterilized bone scissors. The bone pieces were further digested with digesting buffer (3 mg mL^−1^ collagenase I and 4 mg mL^−1^ dispase in PBS) for 30 min at 37 °C and passed through a 40‐µm cell strainer to yield single‐cell suspensions. The products were pooled with bone marrow cells and resuspended in ammonium‐chloride‐potassium (ACK) lysing buffer (Quality Biological, Inc., Gaithersburg, MD) for the lysis of red blood cells. Cells were resuspended in 100‐µL FACS buffer and incubated with allophycocyanin (APC) anti‐mouse F4/80 antibody (1:200, 123116, Biolegend, Inc., San Diego, CA) for 30 min at 4 °C. Cells were then sorted according to green fluorescent protein (GFP) and F4/80 expressions. For analyzing CD68^+^ cells, cells were incubated with APC anti‐mouse F4/80 (1:200, 123116, Biolegend) and phycoerythrin (PE)/Cyanine7 anti‐mouse CD11b (1:200, 101216, Biolegend) for 30 min at 4 °C, followed by fixation and permeabilization. PE anti‐mouse CD68 antibody (1:200, 137014, Biolegend) was then used for CD68 intracellular staining. FACS was performed using a three‐laser cell sorter (BD FACSAria IIu Cell Sorter, BD Biosciences, San Jose, CA) and analyzed with FACSDiva software (version VI, BD Biosciences). Flow cytometry was performed using a BD LSR II flow cytometer (BD Biosciences) and analyzed with FlowJo software (version 10, BD Bioscience).

### µCT Analysis

Mice were euthanized by an overdose of isoflurane inhalation and transcardially perfused with PBS followed by 10% buffered formalin for 5 min. The tibiae were then dissected and fixed in 10% buffered formalin for 24 h and analyzed using a high‐resolution µCT scanner (SkyScan 1275, Bruker, Kontich, Belgium). The scanner was set to a voltage of 65 kV and a current of 153 µA, with a resolution of 9 µm pixel^−1^. Images were reconstructed, analyzed for the diaphyseal cortical bone parameters, and visualized by NRecon version 1.6, CTAn version 1.9, and CTVol version 2.0, software (all, SkyScan), respectively. Before analysis, the proximal TFJ point was aligned with the distal TFJ point along the z‐axis of the scan. Cross‐sectional images of the tibiae were subsequently created to perform 2D analyses of cortical bones. The two regions of interest were defined at 0.9 mm in length centered 5 or 3 mm proximal to the distal TFJ. 2D structural analyses of Ct.Th and B.Ar were collected to represent cortical bone parameters. For finite element analysis, the scanner was set at a resolution of 25 µm pixel^−1^ with the same parameters of voltage and current for bone structural analysis. Bone mineral density (BMD) was calibrated using two phantoms to determine the correlation between Hounsfield unit (HU) and BMD (*ρ*).

### Finite Element Analysis

The geometric data for the mouse tibiae were obtained from µCT images and imported in MIMICS, version 20.0, software (Materialise, Leuven, Belgium) for the segmentation of bone structure. A threshold of 1000 HU was used to distinguish bone from soft tissue. Approximately 337 835 3D elements were used to mesh the bone in 3‐matic, version 12.0, research software (Materialise). The elastic modulus (*E*) of different bone regions was calculated using the equation of Wagner et al.^[^ ^42^ ^]^ Finally, bone elements were assigned as elastic material properties according to HU values where they were located. Boundary conditions were chosen to match the in vivo experimental conditions. The loading axis was defined as the line connecting the proximal TFJ and the geometric center of tibiotalar joint. The distal end of the tibia was completely fixed. Axial loading of 10 N was distributed evenly and applied on the surface of the tibial plateau along the loading axis. The main outcomes of von Mises stress and E33 strains (the strains along the loading axis) on the tibial cortical bone surface were calculated using Abaqus, version 6.9, software (Dassault Systèmes Simulia Corp., Providence, RI).

### Cell Isolation

For the isolation of BMDMs, bone marrow cells were flushed from hindlimbs of 6‐ to 8‐week‐old mice with *α*‐MEM (Gibco, Thermo Fisher Scientific) supplemented with 10% FBS (Gibco, Thermo Fisher Scientific) and 1% penicillin‐streptomycin (Gibco, Thermo Fisher Scientific). The cells were pelleted and seeded in a 10‐cm dish at 37 °C in a 5% CO_2_ humidified incubator overnight. After overnight incubation, the adherent cells were removed, and the non‐adherent cells were collected for BMDM differentiation. The non‐adherent cells were again incubated in the medium with 30 ng mL^−1^ mouse M‐CSF (macrophage‐colony stimulating factor) (Novoprotein, Summit, NJ) to 80% confluence and reseeded for mechanical compression experiments.

To isolate the WPCs, procedures were adapted from a previous study.^[^ ^43^ ^]^ Briefly, hindlimbs were dissected from 6‐ to 8‐week‐old mice. After careful removal of muscle fibers and tendons, the harvested tissue was digested for 10 min at 37 °C with 3 mg mL^−1^ collagenase (C0130, Sigma‐Aldrich) and 4 mg mL^−1^ dispase (D4693, Sigma‐Aldrich). After the digestion, the supernatant was discarded, and the tissue was further digested for an additional 50 min. The released cells were plated on a collagen I‐coated 10‐cm dish and cultured in the medium with 30 ng mL^−1^ mouse M‐CSF for 3 d. The adherent cells were then collected for mechanical compression experiments.

### Flexcell Compression

BMDMs or WPCs (6 × 10^5^ cells/well) were seeded into Matrigel matrix (Corning Incorporated, Corning, NY) with *α*‐MEM medium supplemented with 10% FBS, 1% penicillin‐streptomycin, and 30 ng mL^−1^ mouse M‐CSF in BioPress compression plates (Flexcell International, Burlington, NC) at 37 °C. After allowing the mixture to gel for 30 min, *α*‐MEM medium supplemented with 10% FBS, 1% penicillin‐streptomycin with 30 ng mL^−1^ mouse M‐CSF was added to each well. For BMDMs, following 6 d of incubation, the cyclical compression was applied to the cells in the serum‐free medium by the FlexCell FX‐5000 using pulses of 5, 10, and 20 kPa for 1, 2, 4, and 6 h at a frequency of 0.1 Hz. The cells that underwent inhibitor treatments were cultured in *α*‐MEM medium containing 30 ng mL^−1^ M‐CSF and 1 µM GsMTx4 (R&D Systems, Minneapolis, MN), 5 µM LSKL (Biomatik), or their corresponding vehicles for 24 h before compression loading. For WPCs, after 24 h of incubation, the cyclical compression of 10 kPa was applied to the cells for 1 h per day for 3 d at a frequency of 0.1 Hz. As the control, cells were maintained under the uncompressed condition (0 kPa).

### Histochemistry, Immunohistochemistry, and Histomorphometry

For histochemical and immunohistological analysis, the tibiae were dissected with intact periosteum and fixed in 10% buffered formalin for 24 h. The specimens were then decalcified in 0.5 m ethylenediaminetetraacetic acid (pH 7.4) for 14 d, cut in cross‐sections at 7, 5, or 1 mm proximal to the distal TFJ, and embedded in paraffin or optimal cutting temperature (OCT) compound (Sakura Finetek, Torrance, CA). 4‐µm‐thick transverse‐oriented paraffin sections of tibiae were processed for TRAP (Sigma‐Aldrich) and immunohistochemistry staining using standard protocols. 10‐µm‐thick transverse‐oriented frozen sections of *LysM‐cre::Ai14* mouse tibiae were prepared for immunofluorescent staining using a standard protocol. The sections were incubated with primary antibodies to mouse CD68 (1:500, ab125212, Abcam, Cambridge, UK), mouse F4/80 (1:50, 14‐4801‐82, Thermo Fisher Scientific, Waltham, MA), mouse pSmad2 (1:100, 44‐244G, Thermo Fisher Scientific), mouse osterix (1:200, ab22552, Abcam), mouse osteocalcin (1:200, M188, Takara Bio, Mountain View, CA), and RFP (1:250, 5F8, Chromotek GmbH, Planegg‐Martinsried, Germany) overnight at 4 °C. For immunohistochemical staining, slides were subsequently incubated with secondary antibodies conjugated with horseradish peroxidase for 1 h at room temperature, followed by Chromogenic Substrate (K3468, Dako, Carpinteria, CA) to detect the immunoactivity. The nuclei were counterstained with hematoxylin (H9627, Sigma‐Aldrich). For immunofluorescence staining, slides were incubated with secondary antibodies conjugated with fluorochrome at room temperature for 1 h and mounted with Antifade Mounting Medium with 4′,6‐diamidino‐2‐phenylindole (DAPI) (H‐1200, Vector Laboratories, Burlingame, CA). The specimen images were observed and captured using an Olympus DP72 microscope (Olympus Scientific Solutions Americas Inc., Waltham, MA) and a Zeiss 780 confocal microscope (Zeiss, Oberkochen, Germany). The number of positively stained cells expressed on the posterior and lateral surfaces of the tibial periosteum for each section was counted, and four nonsequential sections were counted and averaged for each mouse in each group. Averaged positive cell numbers were used for the statistical analysis. The adjacent periosteal bone surfaces were quantified in ImageJ, version 1.51d, software (National Institutes of Health, Bethesda, MD) to calculate the number of positively stained cells per millimeter of the periosteal bone surface. For dynamic histomorphometry analysis, the tibiae were dissected and fixed in 70% ethanol for 24 h. The specimens were embedded in methylmethacrylate without decalcification. 300‐µm‐thick transverse‐oriented tibiae sections at 5 or 3 mm proximal to the distal TFJ were obtained, and the sections were polished to 10 µm after mounting on glass slides. The images were captured by a fluorescence microscope. Mineral apposition rate was analyzed using Fiji software at the same region as described above in the histochemical and immunohistological staining section. Loaded tibiae values were normalized to the corresponding non‐loaded tibiae values to determine the changes caused by mechanical loading. The tissue staining procedures and quantitative analysis were performed with the same standard by one investigator blinded to group assignment.

For cellular staining, the cells within the Matrigel matrix were fixed with 4% paraformaldehyde for 30 min after 4 h of compression loading. The primary antibodies to CD68 (1:100, MAB10114, R&D Systems) and F4/80 (1:100, ab6640, Abcam) were then applied to each well overnight at 4 °C. After carefully washing, corresponding secondary antibodies conjugated with fluorochrome were added onto the cells for 1 h and DAPI (H3569, Invitrogen, Thermo Fisher Scientific) for 5 min at room temperature. The viability of the cells was determined using a two‐color fluorescence assay (L3224, Thermo Fisher Scientific) according to the protocol provided by the manufacturer. The cells were then transferred to slides and observed under the confocal microscope.

### RT‐qPCR

Tibiae were collected with intact periosteum free of muscles and tendons. Bone marrow cells were flushed out. The remaining flushed bone was cut into two segments: from 5 mm till 3 mm proximal to the distal TFJ, and from 1 mm proximal to the distal TFJ till the distal end of the tibia. Total RNA was extracted from isolated tibia segments, sorted cells or cultured primary cells using TRIzol reagent (Invitrogen, Thermo Fisher Scientific) according to the manufacturer's instructions. RNA was then reverse transcribed into complementary DNA using the High‐Capacity cDNA Reverse Transcription Kit (4374966, Thermo Fisher Scientific). RT‐qPCR was then performed with Fast SYBR Green Master Mix (4385610, Thermo Fisher Scientific) on QuantStudio 3 Real‐Time PCR System (Thermo Fisher Scientific). Relative expression was calculated for each gene by the 2^−ΔΔCT^ method with actin, beta (*Actb*) as the internal control for normalization. The primers used for each gene were as follows: *Cd68* forward: GAAATGTCACAGTTCACACCAG, reverse: GGATCTTGGACTAGTAGCAGTG; *Adgre1* forward: TGTCTGCATGATCATCACGATA, reverse: CGTGTCCTTGAGTTTAGAGACT; *Piezo1* forward: TCTACTGGCTGTTGCTGCC, reverse: GACCAGCGAGAGAGCATTGA; *Csf1* forward: TTGGATTCTTCTGTGGGGCG, reverse: TGGTGAGGGGTCATAGAATCC; *Tgfb1* forward: GCAACAATTCCTGGCGTTACC, reverse: CAGTGAGCGCTGAATCGAAAG; *Thbs1* forward: CTCCGAGTTGCAAAGGGAGAT, reverse: AAGGACGTTGGTAGAGCTGGA; *Actb* forward: GGAGGGGGTTGAGGTGTT, reverse: GTGTGCACTTTTATTGGTCTCAA.

### ELISA and Western Blot

The primary cells were compressed for 4 h followed by 12 h of uncompressed incubation. The conditioned medium was collected to determine the concentration of total and active TGF‐*β*1 using Mouse TGF‐*β*1 Quantikine ELISA Kit (MB100B, R&D Systems). According to the manufacturer's instructions, active TGF‐*β*1 was measured directly from the conditioned medium, whereas total TGF‐*β*1 was measured from the conditioned medium with acid treatment.

Western blot analyses were conducted on the protein of lysates from the primary cells compressed for 4 h. The supernatants of lysates were collected after centrifugation and separated by sodium dodecyl sulfate‐polyacrylamide gel electrophoresis (SDS‐PAGE), and then blotted on the nitrocellulose blotting membranes (MilliporeSigma, Burlington, MA). The primary antibody for Thbs1 (1:1000, ab267388, Abcam) and *α*‐Actinin (1:1000, 3134, Cell Signaling Technology, Danvers, MA) was applied for incubation, and the proteins were detected using SuperSignal West Femto Maximum Sensitivity Substrate (Thermo Fisher Scientific).

### Statistical Analysis

Statistical analyses were performed using SPSS Statistics, version 24 (IBM Corp., Armonk, NY). The results are presented as mean ± standard error of mean (SEM), unless otherwise noted. For in vivo studies, paired, two‐tailed Student's *t*‐tests were used to determine the loading‐induced morphometric changes at different positions of the tibiae. Unpaired, two‐tailed Student's *t*‐tests were used to analyze sorted cells. Two‐way repeated measures analysis of variance (ANOVA) with Bonferroni post hoc test was performed to test the effect of loading and position, time, genotype, or inhibitor treatment on morphometric changes and different cell or gene expression. For in vitro studies, unpaired, two‐tailed Student's *t*‐tests were used to investigate the changes in gene expression and concentration of TGF‐*β*1 induced by compression. Two‐way ANOVA with Bonferroni post hoc test was used to characterize the effect of compression and inhibitor treatments on gene expression and the concentration of TGF‐*β*1. P values < 0.05 were considered significant. All inclusion/exclusion criteria were preestablished, and no samples or animals were excluded from analyses. No statistical method was used to predetermine the sample size. The experiments were randomized, and analyses were performed by assessors blinded to group assignment.

### Study Approval

The experiment protocols were approved by the Institutional Animal Care and Use Committee of The Johns Hopkins University.

## Conflict of Interest

The authors declare no conflict of interest.

## Supporting information

Supporting Information

## Data Availability

The data that support the findings of this study are available from the corresponding author upon reasonable request.
