# Supplementary material for: Periosteal CD68+F4/80+ Macrophages Are Mechanosensitive for Cortical Bone Formation by Secretion and Activation of TGF‐β1
Source: Adv Sci (Weinh). 2021 Dec 2;9(3):2103343. doi: 10.1002/advs.202103343 (PMC8787385; doi:10.1002/advs.202103343)
Supplement: Supplementary file 1 — Supporting Information [file ADVS-9-2103343-s001.pdf]

ADVANCED SCIENCE
Open Access

Supporting Information

for *Adv. Sci.,* DOI: 10.1002/advs.202103343

Periosteal CD68$^+$F4/80$^+$ Macrophages are Mechanosensitive for Cortical Bone Formation by Secretion and Activation of TGF-β1

*Ruoxian Deng, Changwei Li, Xiao Wang, Leilei Chang, Shuangfei Ni, Weixin Zhang, Peng Xue, Dayu Pan, Mei Wan, Lianfu Deng, Xu Cao**

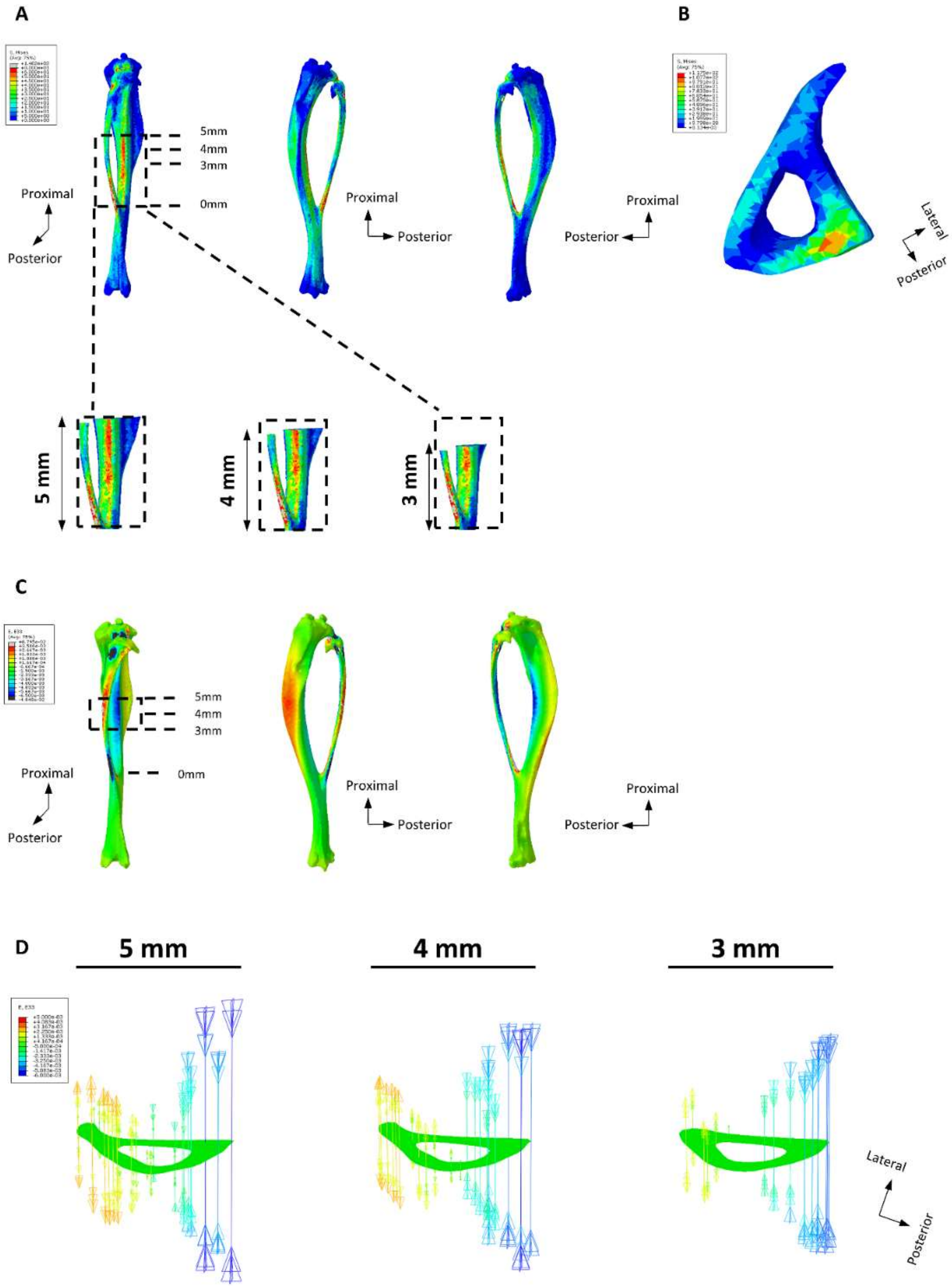

A
B
5mm
4mm
3mm
0mm
Proximal
Posterior
Lateral
5 mm
4 mm
3 mm
C
D

Figure S1. Finite-element analysis of tibia shows the distribution of axial strain along the loading axis. (A) Von Mises stress distribution along the tibial length in three views. Lower panels, higher magnification of the boxed area in the upper panels. (B) Von Mises stress distribution for tibia in the cross-sectional view. (C) The distribution of axial strain (E33) along the tibial length in three views. (D) E33 strain gradient in the cross-sectional view of the boxed area of (C) at 5mm, 4mm and 3mm proximal to the TFJ. Arrows represent the direction of the strain.

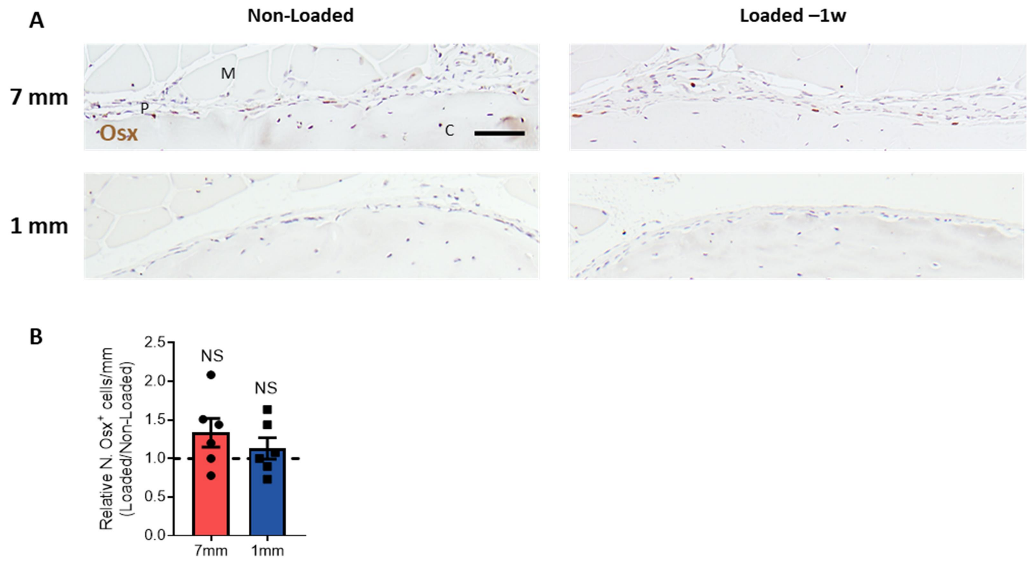

Figure S2. The number of periosteal Osx⁺ cells was not changed at 7 mm or 1 mm proximal to the distal tibiofibular junction (TFJ) after one week of mechanical loading. (A-B) Mice underwent one week of axial compression loading of the tibiae. Non-loaded tibiae were used as controls. (A) Immunohistochemical staining and (B) quantification of Osx^+ cells (brown) on the periosteal tibial surface in WT mice. Scale bar, 50 µm. C, cortical bone; P, periosteum; M, muscle. 1w, one week. The analyses of (A–B) were performed on the cross-sections of posterior and lateral surfaces of tibiae at 7 mm or 1 mm proximal to the distal TFJ. Loaded tibiae values were normalized to the corresponding non-loaded tibiae values. Data are presented as mean ± SEM. $n = 6$ mice. NS, not significant compared with the corresponding non-loaded tibia. Statistical significance was determined by 2-way repeated measures ANOVA with Bonferroni post-hoc test.

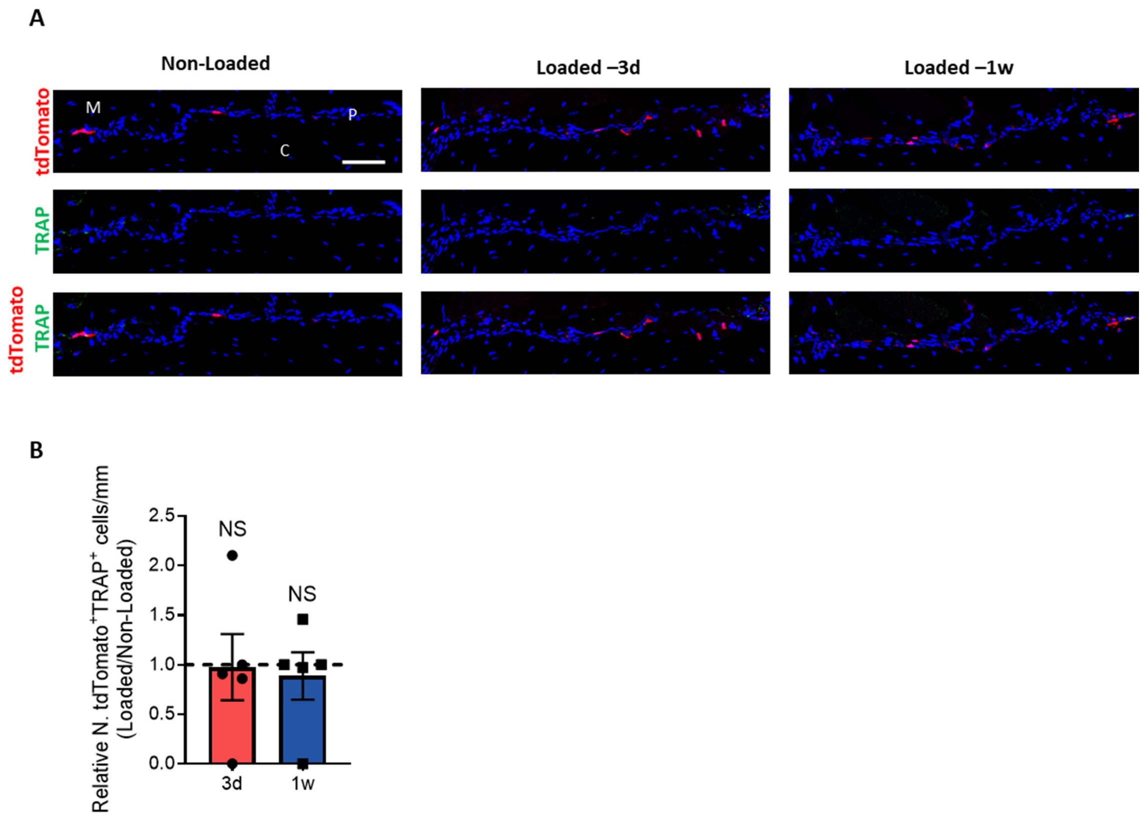

Figure S3. Mechanical loading did not induce the increase in the number of periosteal LysM$^+$TRAP$^+$ cells. (A-B) Mice underwent 3 d or one week of axial compression loading of the tibiae. Non-loaded tibiae were used as controls. (A) Immunohistochemical staining and (B) quantification of tdtomato$^+$ (red) and TRAP$^+$ (green) on the periosteal tibial surface in *LysM-cre::Ai14* mice. Blue indicates 4′,6-diamidino-2-phenylindole (DAPI) staining of nuclei. Scale bar, 50 µm. C, cortical bone; P, periosteum; M, muscle. 3d, 3 days; 1w, one week. The analyses of (A–B) were performed on the cross-sections of posterior and lateral surfaces of tibiae at 5 mm proximal to the distal TFJ. Loaded tibiae values were normalized to the corresponding non-loaded tibiae values. Data are presented as mean ± SEM. $n = 5$ mice at different time points. NS, not significant compared with the corresponding non-loaded tibia. Statistical significance was determined by 2-way repeated measures ANOVA with Bonferroni post-hoc test.

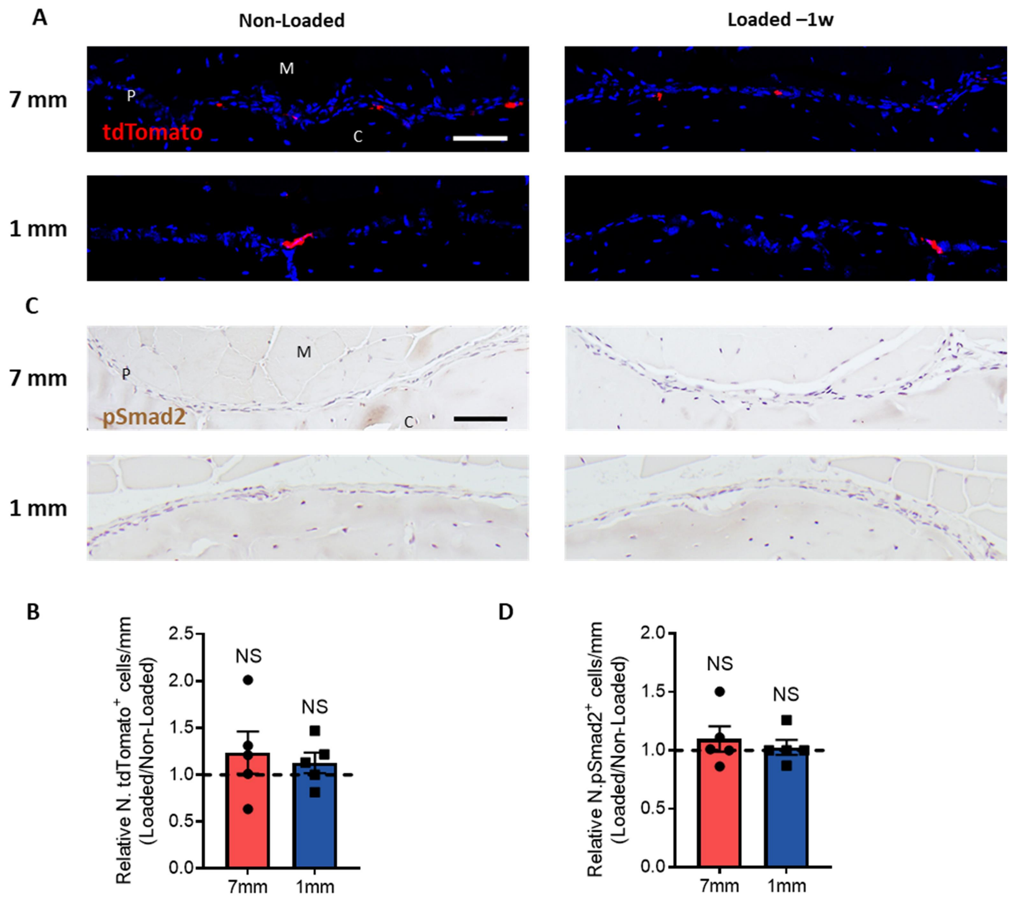

Figure S4. The numbers of periosteal tdTomato$^+$ and pSmad2$^+$ cells were not changed at 7 mm or 1 mm proximal to the distal TFJ after one week of mechanical loading. (A-D) Mice underwent one week of axial compression loading of the tibiae. Non-loaded tibiae were used as controls. (A) Immunofluorescent staining and (B) quantification of tdTomato$^+$ cells (red) on the periosteal tibial surface in *LysM-cre::Ai14* mice. Blue indicates DAPI staining of nuclei. Scale bar, 50 µm. (C) Immunohistochemical staining and (D) quantification of pSmad2$^+$ cells (brown) on the periosteal tibial surface in WT mice. Scale bar, 50 µm. C, cortical bone; P, periosteum; M, muscle. 1w, one week. The analyses of (A–D) were performed on the cross-sections of posterior and lateral surfaces of tibiae at 7 mm or 1 mm proximal to the distal TFJ. Loaded tibiae values were normalized to the corresponding non-loaded tibiae values. Data are presented as mean ± SEM. *n = 5* mice. NS, not significant compared with the corresponding non-loaded tibia. Statistical significance was determined by 2-way repeated measures ANOVA with Bonferroni post-hoc test.

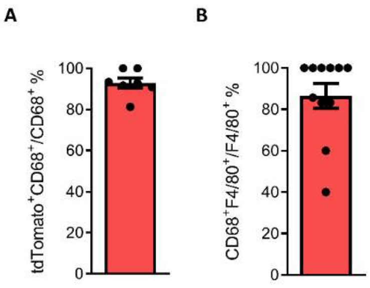

Figure S5. Quantification of immunofluorescent staining of tdTomato$^+$, CD68$^+$ and F4/80$^+$ cells on the periosteal tibial surface. (A) The percentage of CD68$^+$ cells that express tdTomato in *LysM-cre::Ai14* mice (n = *7*). (B) The percentage of F4/80$^+$ cells that express CD68 in WT mice (n = *11*). The analyses of (A & B) were performed on the cross-sectional sections of posterior and lateral surfaces of tibiae at 5 mm proximal to distal TFJ. Data are presented as mean ± SEM.

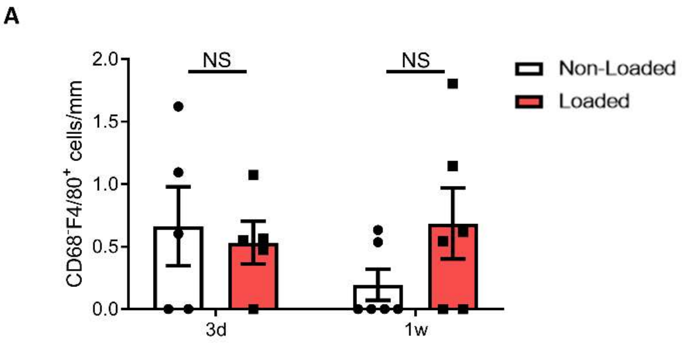

Figure S6. The number of CD68$^-$F4/80$^+$ cells was not changed following mechanical loading. (A) WT mice were subjected to 3 d (n = *5*) or one week (n = *6*) of axial compression loading of tibiae. Non-loaded tibiae used as controls. Quantification of CD68$^-$F4/80$^+$ cells on the periosteal tibial surface. 3d, 3 days; 1w, one week. The analysis of A was performed on the cross-sectional sections of posterior and lateral surfaces of tibiae at 5 mm proximal to distal TFJ. Data are presented as mean ± SEM. NS, not significant compared with the corresponding non-loaded tibia. Statistical significance was determined by two-way repeated measures ANOVA with Bonferroni post-hoc test.

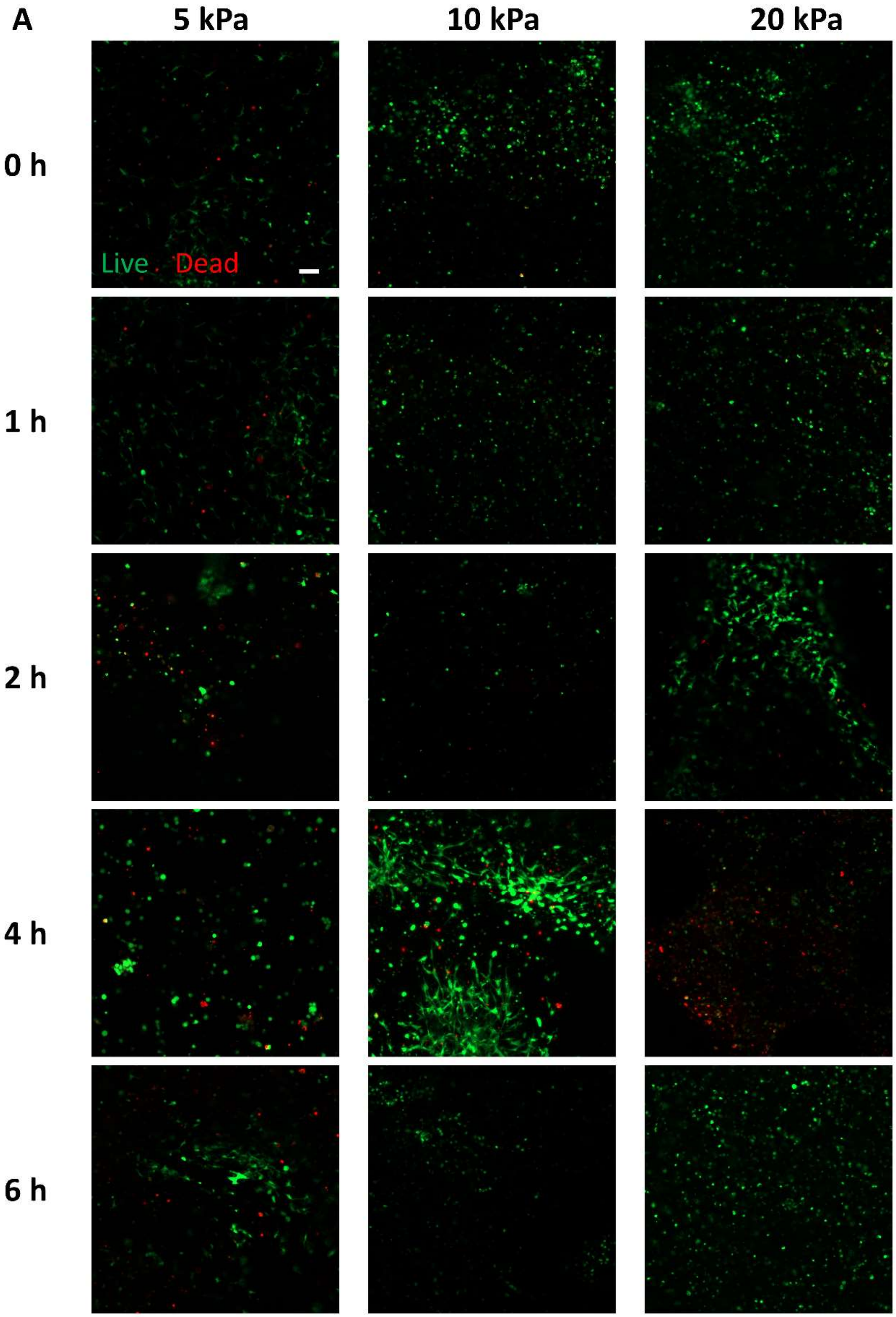
A
5 kPa
10 kPa
20 kPa
0 h
1 h
2 h
4 h
6 h
Live
Dead

Figure S7. Viability staining of BMDMs after mechanical compression. (A) The representative images of LIVE/DEAD viability staining of BMDMs. Primary BMDMs were harvested from WT mice and subjected to 5 kPa, 10 kPa or 20 kPa mechanical compression for 1 h, 2 h, 4 h or 6 h. Cells were maintained under the uncompressed condition (0 kPa as 0 h) as the control. Scale bar, 50 µm.

A

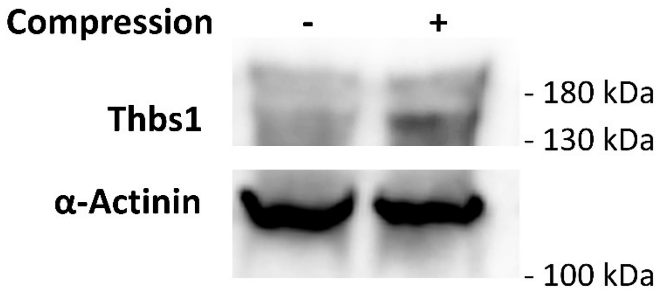

Figure S8. Mechanical compression upregulated the expression of Thbs1 in BMDMs. (A) Western blots of TSP-1 in primary BMDMs. Primary BMDMs were harvested from WT mice and subjected to 10 kPa mechanical compression for 4 h. Cells were maintained under the uncompressed condition as the control. Loading control, α-Actinin.
